# 4-[(*E*)-2-(2-Chloro­benzyl­idene)hydrazin-1-yl]quinolin-1-ium chloride dihydrate

**DOI:** 10.1107/S1600536812022660

**Published:** 2012-05-23

**Authors:** Edward R. T. Tiekink, Solange M. S. V. Wardell, James L. Wardell, Marcelle de Lima Ferreira, Marcus V. N. de Souza, Carlos R. Kaiser

**Affiliations:** a Department of Chemistry, University of Malaya, 50603 Kuala Lumpur, Malaysia; b CHEMSOL, 1 Harcourt Road, Aberdeen AB15 5NY, Scotland; c Centro de Desenvolvimento Tecnológico em Saúde (CDTS), Fundação Oswaldo Cruz (FIOCRUZ), Casa Amarela, Campus de Manguinhos, Av. Brasil 4365, 21040-900 Rio de Janeiro, RJ, Brazil; d Instituto de Tecnologia em Fármacos–Farmanguinhos, FioCruz–Fundação, Oswaldo Cruz, R. Sizenando Nabuco, 100, Manguinhos, 21041-250 Rio de Janeiro, RJ, Brazil; e Programa de Pós-Graduaçõ em Química, Instituto de Química, Universidade Federal do Rio de Janeiro, CP 68563, 21945-970 Rio de Janeiro, Brazil

## Abstract

In the title hydrated salt, C_16_H_13_ClN_3_^+^·Cl^−^·2H_2_O, a small twist is evident in the cation so that the chloro­benzene ring is not coplanar with the central hydrazinyl group [the N—C—C—C torsion angle = −4.8 (12)°]. The conformation about the imine N=C bond [1.284 (10) Å] is *E*. The components of the structure are connected into a three-dimensional architecture *via* O—H⋯O, O—H⋯Cl and N—H⋯Cl hydrogen bonds. One water H atom is disposed over two sites of equal occupancy.

## Related literature

For the biological activity, including the anti-tubercular and anti-tumour activity, of compounds containing the quinolinyl nucleus, see: de Souza *et al.* (2009 ▶); Candea *et al.* (2009 ▶); Montenegro *et al.* (2011 ▶, 2012 ▶). For related structures, see: Howie *et al.* (2010 ▶); de Souza *et al.* (2010 ▶, 2012 ▶); Ferreira *et al.* (2012 ▶); Wardell *et al.* (2012 ▶).
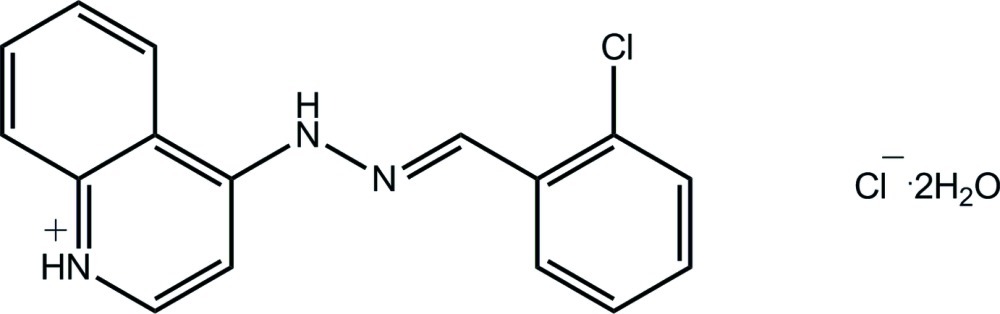


## Experimental

### 

#### Crystal data

C_16_H_13_ClN_3_^+^·Cl^−^·2H_2_O
*M* *_r_* = 354.23
Monoclinic, 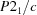

*a* = 4.5946 (3) Å
*b* = 20.1550 (19) Å
*c* = 18.2192 (17) Å
β = 96.660 (5)°
*V* = 1675.8 (2) Å^3^
*Z* = 4
Mo *K*α radiation
μ = 0.40 mm^−1^
*T* = 120 K
0.33 × 0.02 × 0.01 mm

#### Data collection

Bruker–Nonius Roper CCD camera on a κ-goniostat diffractometer
Absorption correction: multi-scan (*SADABS*; Sheldrick, 2007 ▶) *T*_min_ = 0.786, *T*_max_ = 1.000
14027 measured reflections
2924 independent reflections
1565 reflections with *I* > 2σ(*I*)
*R*_int_ = 0.146

#### Refinement

*R*[*F* ^2^ > 2σ(*F* ^2^)] = 0.096
*wR*(*F* ^2^) = 0.223
*S* = 1.06
2924 reflections
229 parameters
11 restraints
H atoms treated by a mixture of independent and constrained refinement
Δρ_max_ = 0.46 e Å^−3^
Δρ_min_ = −0.55 e Å^−3^

### 

Data collection: *COLLECT* (Hooft, 1998 ▶); cell refinement: *DENZO* (Otwinowski & Minor, 1997 ▶) and *COLLECT* (Hooft, 1998 ▶); data reduction: *DENZO* and *COLLECT*; program(s) used to solve structure: *SHELXS97* (Sheldrick, 2008 ▶); program(s) used to refine structure: *SHELXL97* (Sheldrick, 2008 ▶); molecular graphics: *ORTEP-3* (Farrugia, 1997 ▶) and *DIAMOND* (Brandenburg, 2006 ▶); software used to prepare material for publication: *publCIF* (Westrip, 2010 ▶).

## Supplementary Material

Crystal structure: contains datablock(s) global, I. DOI: 10.1107/S1600536812022660/kj2202sup1.cif

Structure factors: contains datablock(s) I. DOI: 10.1107/S1600536812022660/kj2202Isup2.hkl

Supplementary material file. DOI: 10.1107/S1600536812022660/kj2202Isup3.cml

Additional supplementary materials:  crystallographic information; 3D view; checkCIF report

## Figures and Tables

**Table 1:** Hydrogen-bond geometry (Å, °)

*D*—H⋯*A*	*D*—H	H⋯*A*	*D*⋯*A*	*D*—H⋯*A*
N1—H1n⋯Cl2	0.88 (5)	2.32 (5)	3.192 (7)	173 (6)
O1w—H1w⋯Cl2	0.84 (6)	2.37 (6)	3.207 (7)	177 (11)
N2—H2n⋯Cl2^i^	0.88 (6)	2.49 (6)	3.349 (7)	166 (7)
O1w—H2w⋯Cl2^ii^	0.84 (5)	2.42 (7)	3.192 (7)	154 (8)
O2w—H3w⋯O1w^iii^	0.84 (7)	1.96 (7)	2.801 (9)	174 (10)
O2w—H4w⋯O2w^iv^	0.84 (8)	2.08 (10)	2.804 (10)	144 (11)
O2w—H5w⋯O2w^iii^	0.83 (12)	2.05 (13)	2.804 (10)	151 (10)

Symmetry codes: (i) 
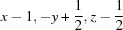
; (ii) 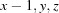; (iii) 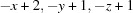; (iv) 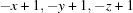.

**Fig. 1.**
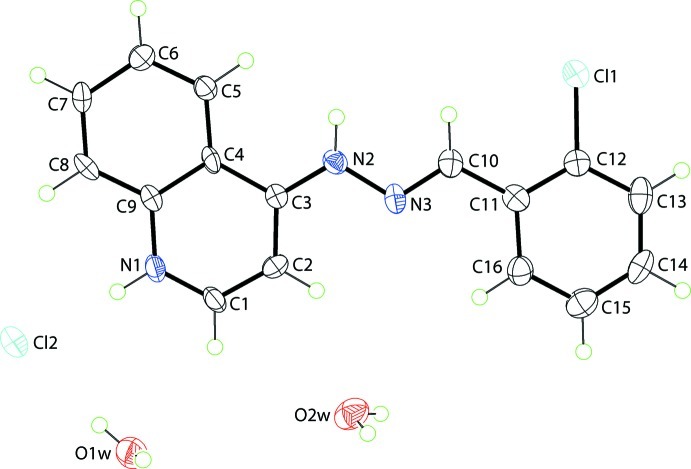
The molecular structure showing the atom-labelling scheme and displacement ellipsoids at the 50% probability level. Only one position of the disordered H atoms of the O2w water molecule is shown.

**Fig. 2.**
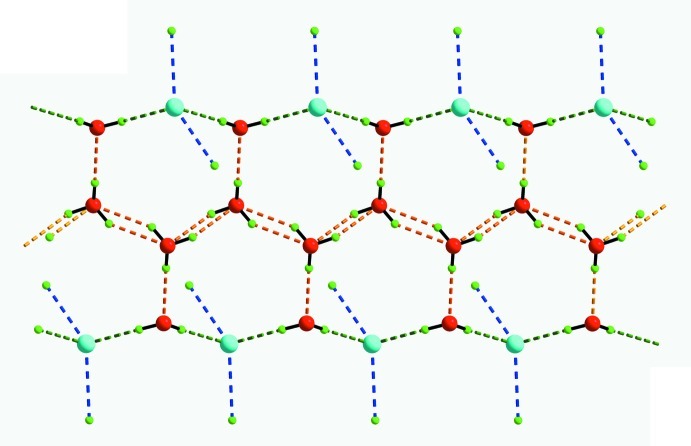
Detail of the hydrogen bonding along the a axis in (I). The O—H···O, O—H···Cl and N—H···Cl hydrogen bonds are shown as orange, green and blue dashed lines, respectively. Only the chloride anions, water molecules and N-bound H atoms are illustrated. The water molecule was disordered with sites of equal weight being resolved for one water-H atom (see text).

**Fig. 3.**
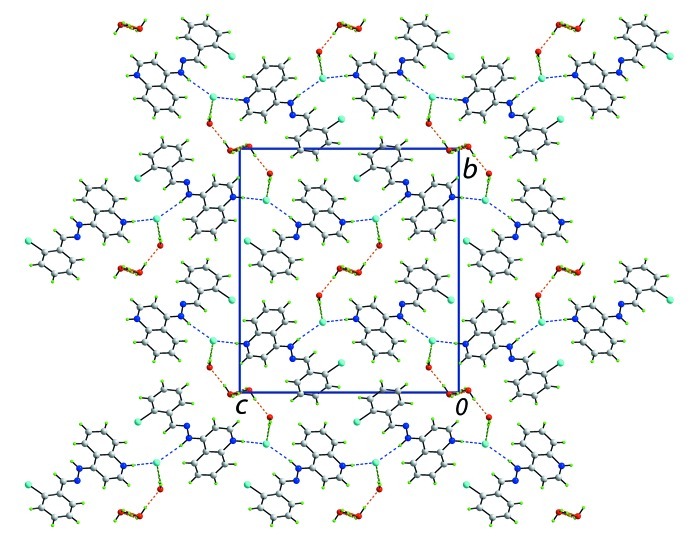
A view in projection down the a axis of the unit-cell contents of (I). The O—H···O, O—H···Cl and N—H···Cl hydrogen bonds are shown as orange, green and blue dashed lines, respectively.

## supplementary crystallographic information

### Comment

A wide range of pharmacological activities have been noted for compounds containing the quinoline nucleus (de Souza *et al.*, 2009), including anti-tubercular (Candea *et al.*, 2009) and anti-tumour (Montenegro *et al.*, 2012) activities. Recently, we have focused attention on arylaldehyde 7-chloroquinoline-4-hydrazone derivatives (Candea *et al.*, 2009; Montenegro *et al.*, 2011). Complementing synthetic studies are crystallographic investigations of these hydrazones (Howie *et al.*, 2010; de Souza *et al.*, 2010; Ferreira *et al.*, 2012; de Souza *et al.*, 2012). We have recently turned our attention to arylaldehyde quinoline-4-hydrazone derivatives (Wardell *et al.*, 2012) and now wish to report the crystal structure of the title hydrated salt, (I).

The asymmetric unit of (I), Fig. 1, comprises a 4-[(*E*)-2-[(2-Chlorophenyl)methylidene]hydrazin-1-yl]quinolin-1-ium cation, a chloride anion and two lattice water molecules. The quinolinyl residue is co-planar with the central hydrazinyl group [the N3—N2—C3—C2 torsional angle is -1.3 (11)°], but the chlorobenzene ring is slightly twisted out of this plane [N3—C10—C11—C16 = -4.8 (12)°]. The conformation about the N3═C10 bond [1.284 (10) Å] is *E*. The molecular structure of (I) resembles very closely that of the 2,4-dichloro analogue (Wardell *et al.*, 2012).

The crystal packing in (I) is dominated by hydrogen bonding interactions, Table 1. The water molecules aggregate into chains along the *a* axis, Fig. 2. One of the O2w—H atoms forms a hydrogen bond to the O1w—O atom. The remaining H atom on O2w is disordered over two positions of equal weight. These interact with adjacent O2w-water molecules as shown in Fig. 2. The two H atoms of O1w form hydrogen bonds with translationally related Cl anions, Fig. 2. Finally, each Cl anion is connected in turn to two quinolinium-H atoms to connect the components of (I) into a three-dimensional architecture, Fig. 3.

### Experimental

A solution of 4-hydrazinoquinoline hydrochloride 1 (1.03 mmol) and 2-chlorobenzaldehyde 2 (1.24 mmol) in ethanol (5 ml) was stirred for 8 h at room temperature and then rotary evaporated. The solid residue was washed with cold Et_2_O (3 \x 10 ml), and recrystallized from EtOH to give the title salt as a dihydrate; *M*.pt 554–555 K. ^1^H NMR (400 MHz, DMSO-d_6_) δ: 14.38 (ls, 1H, NH), 13.07 (ls, 1H, NH), 9.29 (s, 1H, H3'), 8.87 (d, J = 8.4 Hz, 1H, H5), 8.72 (d, J = 6.8 Hz, 1H, H2), (t, J = 7.7 Hz, 1H, H7'), 8.11 (d, J = 8.4 Hz, 1H, H8), 8.04 (t, J = 8.4 Hz, 1H, H7), 7.83 (t, J = 8.4 Hz, 1H, H6), 7.71 (d, J = 6.8 Hz, 1H, H3), 7.60 (d, J = 7.3 Hz, 1H, H8'), 7.57 – 7.47 (m, 2H, H6' and H9').

### Refinement

The C-bound H atoms were geometrically placed (C—H = 0.95 Å) and refined as riding with *U*_iso_(H) = 1.2*U*_eq_(C). The N-bound and O-bound H-atoms were located in a difference Fourier map and refined with a O—H = 0.84±0.01 Å [*U*_iso_(H) = 1.5*U*_eq_(O)] and N—H = 0.88±0.01 Å [*U*_iso_(H) = 1.2*U*_eq_(N)]. One of the O2w—H H atoms was found to be disordered over two sites of equal occupancy with each involved in a significant hydrogen bonding interaction. While the structure has been determined unambiguously, the authors acknowledge that the structure determined is not optimal as seen, for example, in the poor precision in the C—C bonds.

### Crystal data

**Table:**

C_16_H_13_ClN_3_^+^·Cl^−^·2H_2_O	*F*(000) = 736
*M**_r_* = 354.23	*D*_x_ = 1.404 Mg m^−^^3^
Monoclinic, *P*2_1_/*c*	Mo *K*α radiation, λ = 0.71073 Å
Hall symbol: -P 2ybc	Cell parameters from 20901 reflections
*a* = 4.5946 (3) Å	θ = 2.9–27.5°
*b* = 20.1550 (19) Å	µ = 0.40 mm^−^^1^
*c* = 18.2192 (17) Å	*T* = 120 K
β = 96.660 (5)°	Needle, colourless
*V* = 1675.8 (2) Å^3^	0.33 × 0.02 × 0.01 mm
*Z* = 4	

### Data collection

**Table:**

Bruker–Nonius Roper CCD camera on a κ-goniostat diffractometer	2924 independent reflections
Radiation source: Bruker–Nonius FR591 rotating anode	1565 reflections with *I* > 2σ(*I*)
Graphite monochromator	*R*_int_ = 0.146
Detector resolution: 9.091 pixels mm^-1^	θ_max_ = 25.0°, θ_min_ = 3.2°
φ and ω scans	*h* = −5→5
Absorption correction: multi-scan (*SADABS*; Sheldrick, 2007)	*k* = −23→23
*T*_min_ = 0.786, *T*_max_ = 1.000	*l* = −21→21
14027 measured reflections	

### Refinement

**Table:**

Refinement on *F*^2^	Primary atom site location: structure-invariant direct methods
Least-squares matrix: full	Secondary atom site location: difference Fourier map
*R*[*F*^2^ > 2σ(*F*^2^)] = 0.096	Hydrogen site location: inferred from neighbouring sites
*wR*(*F*^2^) = 0.223	H atoms treated by a mixture of independent and constrained refinement
*S* = 1.06	*w* = 1/[σ^2^(*F*_o_^2^) + (0.*P*)^2^ + 18.2957*P*] where *P* = (*F*_o_^2^ + 2*F*_c_^2^)/3
2924 reflections	(Δ/σ)_max_ < 0.001
229 parameters	Δρ_max_ = 0.46 e Å^−^^3^
11 restraints	Δρ_min_ = −0.55 e Å^−^^3^

### Special details

**Table:**

Geometry. All s.u.'s (except the s.u. in the dihedral angle between two l.s. planes) are estimated using the full covariance matrix. The cell s.u.'s are taken into account individually in the estimation of s.u.'s in distances, angles and torsion angles; correlations between s.u.'s in cell parameters are only used when they are defined by crystal symmetry. An approximate (isotropic) treatment of cell s.u.'s is used for estimating s.u.'s involving l.s. planes.
Refinement. Refinement of *F*^2^ against ALL reflections. The weighted *R*-factor *wR* and goodness of fit *S* are based on *F*^2^, conventional *R*-factors *R* are based on *F*, with *F* set to zero for negative *F*^2^. The threshold expression of *F*^2^ > 2σ(*F*^2^) is used only for calculating *R*-factors(gt) *etc*. and is not relevant to the choice of reflections for refinement. *R*-factors based on *F*^2^ are statistically about twice as large as those based on *F*, and *R*- factors based on ALL data will be even larger.

### Fractional atomic coordinates and isotropic or equivalent isotropic displacement parameters (Å^2^)

**Table:**

	*x*	*y*	*z*	*U*_iso_*/*U*_eq_	Occ. (<1)
Cl1	0.2444 (5)	0.37879 (12)	0.03537 (12)	0.0356 (6)	
N1	1.3893 (15)	0.3016 (3)	0.4691 (4)	0.0277 (17)	
H1N	1.492 (15)	0.299 (4)	0.513 (2)	0.033*	
N2	0.8798 (14)	0.3126 (3)	0.2682 (4)	0.0242 (15)	
H2N	0.891 (17)	0.283 (3)	0.233 (3)	0.029*	
N3	0.6721 (14)	0.3622 (3)	0.2585 (4)	0.0268 (16)	
C1	1.1941 (17)	0.3493 (4)	0.4555 (4)	0.0269 (19)	
H1	1.1704	0.3806	0.4934	0.032*	
C2	1.0242 (17)	0.3555 (4)	0.3889 (4)	0.0248 (19)	
H2	0.8891	0.3912	0.3809	0.030*	
C3	1.0490 (16)	0.3096 (4)	0.3328 (4)	0.0202 (17)	
C4	1.2699 (17)	0.2574 (4)	0.3463 (4)	0.0230 (18)	
C5	1.3246 (16)	0.2098 (4)	0.2931 (4)	0.0226 (18)	
H5	1.2159	0.2107	0.2455	0.027*	
C6	1.5348 (18)	0.1623 (4)	0.3100 (4)	0.0278 (19)	
H6	1.5708	0.1303	0.2739	0.033*	
C7	1.6980 (17)	0.1602 (4)	0.3803 (4)	0.0265 (19)	
H7	1.8415	0.1266	0.3914	0.032*	
C8	1.6507 (17)	0.2062 (4)	0.4325 (4)	0.0252 (19)	
H8	1.7631	0.2054	0.4796	0.030*	
C9	1.4343 (17)	0.2546 (4)	0.4157 (4)	0.0247 (19)	
C10	0.5259 (16)	0.3655 (4)	0.1942 (5)	0.0259 (19)	
H10	0.5643	0.3353	0.1565	0.031*	
C11	0.3006 (18)	0.4161 (4)	0.1795 (4)	0.0263 (19)	
C12	0.1484 (17)	0.4252 (4)	0.1102 (4)	0.0257 (19)	
C13	−0.0717 (18)	0.4732 (4)	0.0953 (5)	0.034 (2)	
H13	−0.1737	0.4784	0.0472	0.041*	
C14	−0.1342 (18)	0.5130 (4)	0.1541 (5)	0.033 (2)	
H14	−0.2819	0.5460	0.1458	0.040*	
C15	0.0101 (19)	0.5057 (4)	0.2227 (5)	0.036 (2)	
H15	−0.0375	0.5336	0.2616	0.043*	
C16	0.2242 (18)	0.4586 (4)	0.2365 (5)	0.030 (2)	
H16	0.3232	0.4543	0.2850	0.036*	
Cl2	1.8077 (5)	0.28801 (11)	0.62092 (11)	0.0351 (6)	
O1W	1.3131 (14)	0.3975 (3)	0.6374 (4)	0.0408 (16)	
H1W	1.446 (13)	0.369 (3)	0.635 (6)	0.061*	
H2W	1.150 (9)	0.379 (4)	0.640 (6)	0.061*	
O2W	0.7300 (15)	0.4920 (3)	0.4565 (4)	0.0455 (17)	
H3W	0.72 (2)	0.527 (3)	0.431 (5)	0.068*	
H4W	0.563 (12)	0.484 (7)	0.469 (8)	0.068*	0.50
H5W	0.85 (3)	0.495 (7)	0.494 (5)	0.068*	0.50

### Atomic displacement parameters (Å^2^)

**Table:**

	*U*^11^	*U*^22^	*U*^33^	*U*^12^	*U*^13^	*U*^23^
Cl1	0.0366 (12)	0.0402 (13)	0.0283 (11)	0.0061 (11)	−0.0034 (9)	−0.0028 (10)
N1	0.031 (4)	0.028 (4)	0.022 (4)	0.004 (3)	−0.007 (3)	0.003 (3)
N2	0.026 (4)	0.024 (4)	0.022 (4)	−0.002 (3)	−0.001 (3)	−0.002 (3)
N3	0.024 (4)	0.027 (4)	0.027 (4)	0.000 (3)	−0.005 (3)	0.005 (3)
C1	0.030 (5)	0.035 (5)	0.015 (4)	0.001 (4)	−0.002 (4)	0.002 (4)
C2	0.027 (4)	0.018 (4)	0.030 (5)	0.002 (4)	0.005 (4)	−0.002 (4)
C3	0.020 (4)	0.020 (4)	0.020 (4)	−0.003 (3)	0.001 (3)	0.002 (3)
C4	0.028 (4)	0.023 (5)	0.016 (4)	0.000 (4)	−0.004 (3)	0.008 (3)
C5	0.021 (4)	0.028 (5)	0.019 (4)	0.000 (4)	0.002 (3)	0.001 (4)
C6	0.031 (5)	0.022 (5)	0.030 (5)	0.002 (4)	0.003 (4)	0.000 (4)
C7	0.024 (4)	0.029 (5)	0.025 (4)	0.004 (4)	−0.002 (3)	0.010 (4)
C8	0.027 (4)	0.035 (5)	0.013 (4)	−0.007 (4)	−0.003 (3)	0.001 (4)
C9	0.027 (4)	0.029 (5)	0.017 (4)	0.000 (4)	−0.002 (3)	0.004 (4)
C10	0.023 (4)	0.021 (5)	0.032 (5)	−0.005 (4)	−0.002 (4)	0.001 (4)
C11	0.031 (5)	0.022 (5)	0.025 (4)	−0.006 (4)	0.002 (4)	0.002 (4)
C12	0.026 (4)	0.023 (5)	0.028 (5)	−0.003 (4)	0.003 (4)	0.001 (4)
C13	0.026 (5)	0.030 (5)	0.045 (6)	−0.003 (4)	−0.004 (4)	0.013 (4)
C14	0.024 (4)	0.024 (5)	0.052 (6)	0.006 (4)	0.006 (4)	0.008 (4)
C15	0.038 (5)	0.029 (5)	0.042 (6)	0.002 (4)	0.010 (5)	0.001 (4)
C16	0.024 (4)	0.034 (5)	0.032 (5)	0.000 (4)	0.002 (4)	0.002 (4)
Cl2	0.0372 (12)	0.0408 (13)	0.0258 (11)	−0.0028 (11)	−0.0026 (9)	0.0033 (10)
O1W	0.043 (4)	0.046 (4)	0.034 (4)	0.001 (3)	0.009 (3)	0.006 (3)
O2W	0.057 (4)	0.033 (4)	0.049 (4)	0.009 (4)	0.018 (4)	0.007 (3)

### Geometric parameters (Å, º)

**Table:**

Cl1—C12	1.752 (8)	C7—H7	0.9500
N1—C1	1.319 (10)	C8—C9	1.401 (11)
N1—C9	1.390 (10)	C8—H8	0.9500
N1—H1N	0.882 (10)	C10—C11	1.456 (11)
N2—C3	1.335 (9)	C10—H10	0.9500
N2—N3	1.380 (9)	C11—C12	1.382 (11)
N2—H2N	0.880 (10)	C11—C16	1.421 (12)
N3—C10	1.284 (10)	C12—C13	1.404 (11)
C1—C2	1.371 (10)	C13—C14	1.393 (12)
C1—H1	0.9500	C13—H13	0.9500
C2—C3	1.392 (10)	C14—C15	1.353 (12)
C2—H2	0.9500	C14—H14	0.9500
C3—C4	1.463 (11)	C15—C16	1.369 (12)
C4—C9	1.398 (10)	C15—H15	0.9500
C4—C5	1.406 (11)	C16—H16	0.9500
C5—C6	1.370 (11)	O1W—H1W	0.842 (10)
C5—H5	0.9500	O1W—H2W	0.840 (10)
C6—C7	1.407 (11)	O2W—H3W	0.841 (10)
C6—H6	0.9500	O2W—H4W	0.841 (11)
C7—C8	1.363 (11)	O2W—H5W	0.840 (10)
			
C1—N1—C9	121.3 (7)	C9—C8—H8	120.4
C1—N1—H1N	120 (6)	N1—C9—C4	119.8 (7)
C9—N1—H1N	119 (6)	N1—C9—C8	118.8 (7)
C3—N2—N3	117.9 (6)	C4—C9—C8	121.4 (7)
C3—N2—H2N	123 (5)	N3—C10—C11	119.3 (8)
N3—N2—H2N	119 (5)	N3—C10—H10	120.4
C10—N3—N2	115.8 (7)	C11—C10—H10	120.4
N1—C1—C2	122.5 (8)	C12—C11—C16	116.5 (8)
N1—C1—H1	118.7	C12—C11—C10	122.2 (8)
C2—C1—H1	118.7	C16—C11—C10	121.3 (7)
C1—C2—C3	120.2 (8)	C11—C12—C13	123.0 (8)
C1—C2—H2	119.9	C11—C12—Cl1	119.6 (6)
C3—C2—H2	119.9	C13—C12—Cl1	117.3 (6)
N2—C3—C2	121.9 (7)	C14—C13—C12	117.1 (8)
N2—C3—C4	120.1 (7)	C14—C13—H13	121.4
C2—C3—C4	118.0 (7)	C12—C13—H13	121.4
C9—C4—C5	118.3 (7)	C15—C14—C13	121.6 (8)
C9—C4—C3	118.2 (7)	C15—C14—H14	119.2
C5—C4—C3	123.4 (7)	C13—C14—H14	119.2
C6—C5—C4	120.0 (7)	C14—C15—C16	120.7 (9)
C6—C5—H5	120.0	C14—C15—H15	119.7
C4—C5—H5	120.0	C16—C15—H15	119.7
C5—C6—C7	120.9 (8)	C15—C16—C11	121.1 (8)
C5—C6—H6	119.6	C15—C16—H16	119.5
C7—C6—H6	119.6	C11—C16—H16	119.5
C8—C7—C6	120.2 (8)	H1W—O1W—H2W	111 (6)
C8—C7—H7	119.9	H3W—O2W—H4W	108 (6)
C6—C7—H7	119.9	H3W—O2W—H5W	112 (6)
C7—C8—C9	119.2 (7)	H4W—O2W—H5W	110 (6)
C7—C8—H8	120.4		
			
C3—N2—N3—C10	176.2 (7)	C3—C4—C9—N1	−1.4 (11)
C9—N1—C1—C2	0.3 (13)	C5—C4—C9—C8	0.6 (12)
N1—C1—C2—C3	1.4 (13)	C3—C4—C9—C8	−179.7 (7)
N3—N2—C3—C2	−1.3 (11)	C7—C8—C9—N1	−179.5 (7)
N3—N2—C3—C4	179.2 (7)	C7—C8—C9—C4	−1.2 (12)
C1—C2—C3—N2	177.5 (7)	N2—N3—C10—C11	179.5 (7)
C1—C2—C3—C4	−3.0 (11)	N3—C10—C11—C12	175.7 (8)
N2—C3—C4—C9	−177.5 (7)	N3—C10—C11—C16	−4.8 (12)
C2—C3—C4—C9	2.9 (11)	C16—C11—C12—C13	−0.2 (12)
N2—C3—C4—C5	2.2 (12)	C10—C11—C12—C13	179.3 (8)
C2—C3—C4—C5	−177.3 (7)	C16—C11—C12—Cl1	175.8 (6)
C9—C4—C5—C6	0.0 (12)	C10—C11—C12—Cl1	−4.7 (11)
C3—C4—C5—C6	−179.7 (7)	C11—C12—C13—C14	0.2 (12)
C4—C5—C6—C7	0.0 (12)	Cl1—C12—C13—C14	−175.9 (6)
C5—C6—C7—C8	−0.7 (13)	C12—C13—C14—C15	−0.1 (13)
C6—C7—C8—C9	1.2 (12)	C13—C14—C15—C16	0.0 (14)
C1—N1—C9—C4	−0.2 (12)	C14—C15—C16—C11	0.0 (13)
C1—N1—C9—C8	178.1 (8)	C12—C11—C16—C15	0.1 (12)
C5—C4—C9—N1	178.9 (7)	C10—C11—C16—C15	−179.3 (8)

### Hydrogen-bond geometry (Å, º)

**Table:**

*D*—H···*A*	*D*—H	H···*A*	*D*···*A*	*D*—H···*A*
N1—H1n···Cl2	0.88 (5)	2.32 (5)	3.192 (7)	173 (6)
O1w—H1w···Cl2	0.84 (6)	2.37 (6)	3.207 (7)	177 (11)
N2—H2n···Cl2^i^	0.88 (6)	2.49 (6)	3.349 (7)	166 (7)
O1w—H2w···Cl2^ii^	0.84 (5)	2.42 (7)	3.192 (7)	154 (8)
O2w—H3w···O1w^iii^	0.84 (7)	1.96 (7)	2.801 (9)	174 (10)
O2w—H4w···O2w^iv^	0.84 (8)	2.08 (10)	2.804 (10)	144 (11)
O2w—H5w···O2w^iii^	0.83 (12)	2.05 (13)	2.804 (10)	151 (10)

Symmetry codes: (i) *x*−1, −*y*+1/2, *z*−1/2; (ii) *x*−1, *y*, *z*; (iii) −*x*+2, −*y*+1, −*z*+1; (iv) −*x*+1, −*y*+1, −*z*+1.
